# Low Carbohydrate Availability in Energy Balance Alters Bone Turnover and Muscle Proteomic Response With Limited Endocrine Disruption

**DOI:** 10.1096/fj.202602531R

**Published:** 2026-08-31

**Authors:** Erick Mosquera‐Lopez, Harry L. Taylor, Yusuke Nishimura, Carl Langan‐Evans, Sam O. Shepherd, Juliette A. Strauss, James P. Morton, Jatin G. Burniston, Jose L. Areta

**Affiliations:** ^1^ Research Institute for Sport & Exercise Sciences (RISES) Liverpool John Moores University Liverpool UK; ^2^ Precision Fuel & Hydration Ltd. Dorset UK

**Keywords:** dietary periodisation, energy deficit, low‐carbohydrate high‐fat diet, muscle proteomics

## Abstract

Training with low carbohydrate availability (LCA) has been proposed as an independent determinant of physiological perturbations commonly attributed to low energy availability (LEA) and to increase skeletal muscle oxidative machinery, yet the effects of LCA in isolation from LEA remain unclear. We examined whether short‐term carbohydrate restriction under energy balance alters endocrine and metabolic markers associated with LEA and skeletal muscle proteomic response. In a randomized crossover design, eight trained males completed 4 days of either a low‐carbohydrate high‐fat diet (LOW; 12% carbohydrate, 69% fat, 19% protein) or a normal‐carbohydrate diet (NORM; 62% carbohydrate, 19% fat, 19% protein), while undertaking daily cycloergometer exercise (15 kcal kg FFM^−1^ day^−1^) and maintaining energy availability at 45 kcal kg FFM^−1^ day^−1^. LOW induced a clear metabolic shift consistent with LCA, evidenced by elevated circulating free fatty acids, glycerol and β‐hydroxybutyrate, in fasting conditions and fat oxidation at rest and during exercise, alongside reduced exercise glucose concentrations. Despite these responses, LOW did not alter insulin, testosterone, triiodothyronine, leptin, hepcidin, or P1NP. In contrast, β‐CTX increased and IGF‐1 decreased relative to NORM. Muscle glycogen concentration decreased only in LOW (40% ± 14%). Proteomic analysis identified 671 proteins; 57 differentially expressed in LOW relative to NORM were limited to fatty acid metabolism pathways and suppression of ribosomal, sarcomeric, and extracellular matrix proteins. These findings indicate that isolated LCA exerts limited endocrine disruption but may selectively compromise bone turnover and muscle anabolic response, suggesting that without acute LEA, LCA has limited influence on muscle oxidative phenotype.

## Graphical abstract

Eight trained males completed 4 days of normal‐carbohydrate or low‐carbohydrate (81% reduction in carbohydrate) diets while maintaining energy balance and undertaking daily exercise. Low carbohydrate decreased total body water and fat‐free mass, increased circulating free fatty acids, glycerol and β‐hydroxybutyrate, alongside increased fat oxidation at rest and during exercise, but with minor endocrine disruption. However, low carbohydrate availability increased β‐CTX, decreased IGF‐1, reduced skeletal muscle glycogen, and altered skeletal muscle proteins involved in lipid metabolism, translation, sarcomeric and extracellular matrix.

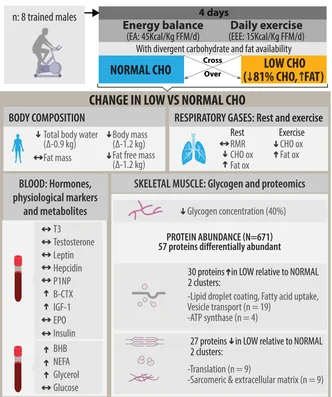

## 1 Introduction

Whether endocrine and metabolic disturbances commonly attributed to low energy availability (LEA) arise from energy deficiency itself or from concomitant reductions in carbohydrate availability remains unresolved. LEA is recognized as a potent modulator of endocrine, metabolic, and physiological function in exercising individuals [1] and it is widely regarded as the principal etiological factor underpinning theoretical frameworks such as Relative Energy Deficiency in Sport syndrome (REDs) and the Male and Female Athlete Triad [2, 3, 4]. However, the current evidence supporting these models remains largely observational, limiting causal inference and complicating attribution of physiological outcomes specifically to LEA [5].

Carbohydrate availability is a particularly important confounder because reductions in energy intake are typically accompanied by marked reductions in carbohydrate intake [1, 6]. Consequently, responses observed during LEA may reflect low carbohydrate availability (LCA), reduced energy availability per se, or an interaction between the two. Importantly, LCA may also occur independently of LEA, such as during low‐carbohydrate high‐fat diets which maintain overall energy balance.

Mechanistically, LCA has been proposed to influence both central and peripheral pathways implicated in LEA‐related responses. Given the brain's dependence on glucose in the absence of ketone bodies and the presence of glucose‐sensing neurons linked to major endocrine axes [6, 7], reduced carbohydrate availability may contribute to endocrine alterations frequently attributed to LEA. LCA has been linked to suppression of testosterone and triiodothyronine (T3) [8], yet direct experimental support for these central effects remains limited. By contrast, peripheral effects of LCA—including altered bone turnover, iron metabolism, skeletal muscle glycogen depletion, and modified training adaptation—are better supported by experimental and observational data [9, 10, 11, 12, 13, 14, 15], although most studies have been conducted in race‐walkers during intensified competition preparation or in females only.

Experimental LEA research has historically focused on females because of the high prevalence of menstrual dysfunction and bone stress injuries in that population [1]. Consequently, research on the effect of LEA and LCA on males is limited. Recently, we reported one of the few comprehensive experimental investigations of LEA in exercising males to date, demonstrating endocrine responses broadly consistent with those observed in females following experimental manipulation of energy availability. However, carbohydrate intake in that study was simultaneously reduced by ~65% (from 6.7 to 2.4 g kg^−1^ day^−1^) resulting in a reduction of muscle glycogen of 28% [16], precluding clear distinction between effects attributable to LEA and those attributable to LCA.

Importantly, in addition to modulating the endocrine milieu, LCA has been implicated in promoting several skeletal muscle training adaptations related to fat oxidation and mitochondrial biogenesis through intracellular signaling related to low muscle glycogen concentration [10, 17, 18, 19]. However, our recent work demonstrated that short‐term (5 days) low energy availability (LEA), when overlayed on top of the exercise stimulus, induces a robust and coordinated upregulation of mitochondrial proteins within the skeletal muscle proteome compared to energy balance conditions [16]. In this context, those findings suggest that acute energy deficit, rather than carbohydrate availability per se, may be a primary driver of mitochondrial remodeling in this context. This distinction is important, as LCA and LEA frequently co‐occur in applied settings, making it difficult to disentangle their independent contributions to skeletal muscle adaptation. Given that carbohydrate periodisation remains a central strategy in exercise nutrition with direct implications for training adaptation and performance [20], clarifying the specific role of carbohydrate availability in regulating skeletal muscle phenotype represents a key priority for future research.

Therefore, the present study examined the effects of reduced carbohydrate availability during energy balance on endocrine, metabolic, and physiological markers commonly associated with LEA in exercising males, with a comprehensive skeletal muscle proteomic analysis.

We hypothesized that LCA would reduce muscle glycogen, and circulating concentrations of triiodothyronine (T3), leptin, P1NP, insulin‐like growth factor‐1 (IGF‐1), glucose, and insulin; increase β‐CTx, hepcidin, plasma glycerol, non‐esterified fatty acids (NEFA), and ketone bodies; and leave total testosterone and resting metabolic rate unchanged while retooling skeletal muscle proteome towards a more oxidative phenotype.

## 2 Methods

### 2.1 Participants

We recruited 8 trained, healthy, non‐smoking males (age: 27 ± 4 (means ± SD); height: 1.79 ± 0.06 m; body mass: 79 ± 12 kg; body fat: 17% ± 4%; V̇O_2max_: 54 ± 6 mL kg^−1^ min^−1^; peak power output (PPO): 323 ± 43 W) who regularly completed ≥ 3 endurance‐based exercise sessions week^−1^ and were classified as Tier 1–2 participants [21]. The study was approved by the local Research Ethics Committee of Liverpool John Moores University (ref. no. H22/SPS/026), and the participants provided written consent prior to participation.

### 2.2 General Study Design

**FIGURE 1:**
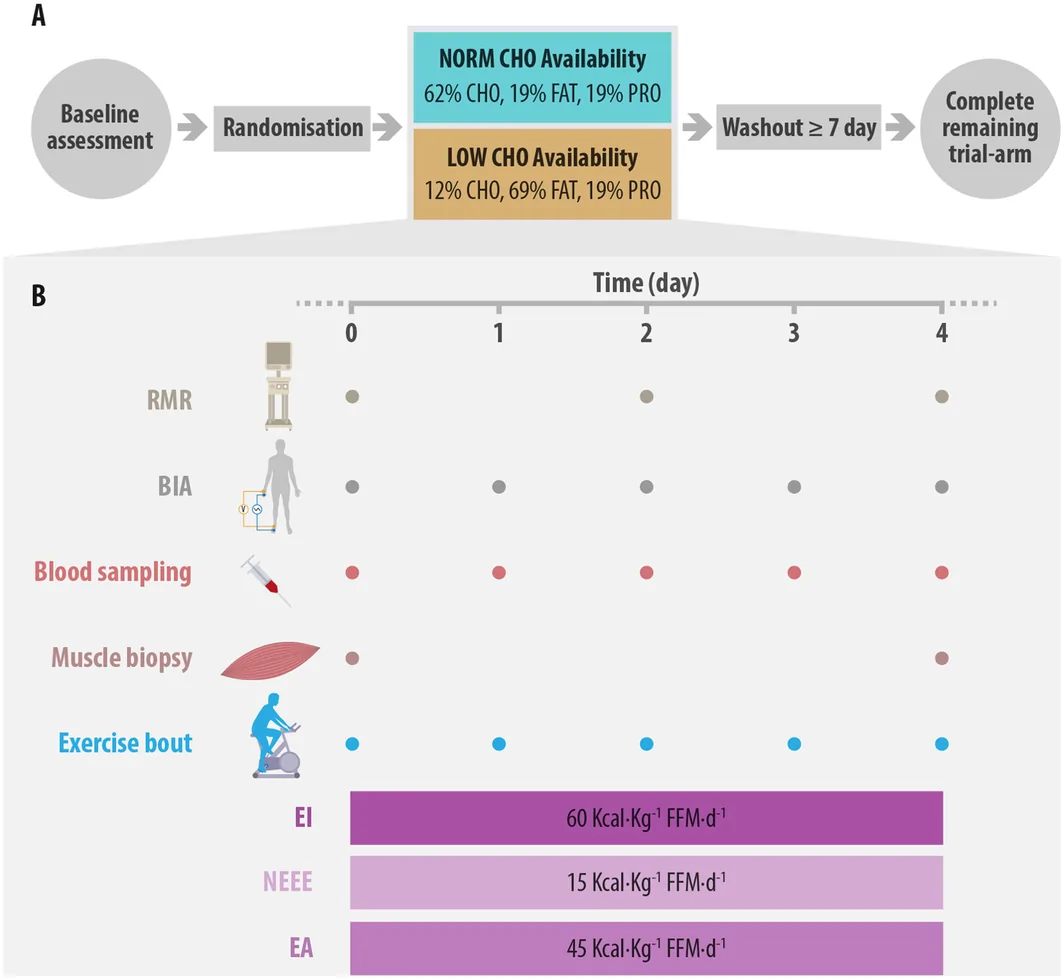
Schematic overview of the study design. Participants completed two 5‐day experimental interventions in a randomized order, and counterbalanced, each finishing in the morning of Day 4, after morning exercise. Interventions were separated by a ≥ 7‐day washout period. Between Days 0 and 3, inclusive, participants consumed custom‐made prepackaged diets providing either normal (NORM) or low (LOW) carbohydrate availability. From Day 0 to Day 4, participants completed supervised endurance‐based exercise bouts in the morning. BIA, bio‐electrical impedance analysis; EA, energy availability; EI, energy intake; NEEE, net exercise energy expenditure; RMR, resting metabolic rate assessment.

An overview of the study design is shown in Figure 1. Using a randomized, counterbalanced, crossover study design, participants completed baseline testing, followed by two separate study intervention periods of four consecutive days of either normal (NORM) or low (LOW) carbohydrate availability (see Table 1 for diet details) with daily cycloergometry exercise set to expend 15 kcal kg FFM^−1^ at 95% of Lactate Turnpoint 1 (LTP1). A washout period ≥ 7 days was implemented between trials.

**TABLE 1:** Energy and macronutrient intake for NORM and LOW.

	NORM	LOW
Energy intake		
Absolute EI (kcal)	3927 ± 460	3924 ± 458
Relative EI (kcal kg FFM^−1^ day^−1^)	60 ± 0	60 ± 0
CHO intake		
Energy from CHO (%)	62 ± 1	12 ± 0
CHO (g day^−1^)	607 ± 73	117 ± 15
CHO (g kg^−1^ day^−1^)	7.7 ± 0.4	1.5 ± 0.0
FAT intake		
Energy from FAT (%)	19 ± 1	69 ± 1
FAT (g day^−1^)	84 ± 8	301 ± 35
FAT (g kg^−1^ day^−1^)	1.1 ± 0.1	3.8 ± 0.2
PRO intake		
Energy from PRO (%)	19 ± 1	19 ± 1
PRO (g day^−1^)	185 ± 25	186 ± 24
PRO (g kg^−1^ day^−1^)	2.4 ± 0.1	2.4 ± 0.1

*Note:* Values are means ± SD (*n* = 8).

### 2.3 Baseline Testing

Participants attended the laboratory in an overnight fasted state for a baseline test 2 to 7 days prior to the study intervention. Body composition was assessed with multifrequency octopolar bio‐electrical impedance (BIA) (SECA mBCA 515: SECA GMBH, Hamburg, Germany) followed by a two‐part incremental cycle test (Lode Corival cpet: Lode, Groningen, Netherlands) to determine LTP 1, sub‐maximal (V̇O_2_), and maximal oxygen consumption (V̇O_2max_). For part one, participants cycled at 90 W for 3 min with 30 W increments every 3 min until LTP 1 was achieved, which was defined as the exercise intensity where blood lactate concentrations increased by ≥ 1.0 mM across the 3 min stage [22]. Respiratory gases (Moxus modular metabolic system, AEI technologies, Pittsburgh, PA, USA), heart rate (HR, H7 Polar, Polar Electro Oy, Kempele, Finland), and rating of perceived exertion (RPE, 6–20 scale, [23]) were recorded during the final 30 s of each stage, and capillary blood samples were also collected to measure lactate concentrations (Biosen C‐Line, EKF Diagnostics, Cardiff, UK). For part two, participants underwent an incremental cycling test to exhaustion [22]. A test was considered maximal if at least two of the following criteria were attained: (i) HR within 10 beats min^−1^of age predicted maximum, (ii) respiratory exchange ratio (RER) > 1.1, and (iii) V̇O_2_ plateau despite increased workload. V̇O_2max_ was determined as the highest 30 s average value attained during the exercise test. After ≥ 20 min rest, participants cycled for 3 min at 125 W and 17 min at 95% LTP 1 for familiarization.

### 2.4 Experimental Sessions

Before each experimental session, on Day −4, participants body composition was assessed with BIA (SECA mBCA 515; SECA GMBH, Hamburg, Germany). From Day 0 to Day 4, participants arrived at the laboratory (0700 to 0800) in a fasted condition and having minimized active transport to arrive at the facilities. Participants completed in chronological order (i) resting metabolic rate (RMR) assessment—Days 0, 2 and 4 only, (ii) body composition assessment, (iii) venous blood sampling, (iv) skeletal muscle biopsy—Days 0 and 4 only, (v) cycling exercise at 95% LTP 1 until achieving a net exercise energy expenditure (NEEE) equating 15 kcal kg FFM^−1^, (vi) dietary consumption throughout the rest of the day (Days 0 through to 3, inclusive) of a NORM or LOW CHO meal plan providing an energy intake (EI) of 60 kcal kg FFM^−1^ day^−1^ to achieve an energy availability (EA) of 45 kcal kg FFM^−1^ day^−1^ for both experimental sessions.

### 2.5 Energy and Carbohydrate Availability Calculations

The fat‐free mass (FFM) recorded through BIA on Day −4 served for the calculation of the dietary provision and NEEE for each experimental session. NEEE was determined by subtracting the RMR from gross exercise energy expenditure (GEEE). GEEE was calculated from V̇O_2_ and V̇CO_2_ [24]. The EA equation was defined as [EA = (EI–NEEE)/FFM] [25]. Carbohydrate availability was operationally defined as the difference between the daily carbohydrate ingested and carbohydrate oxidation during exercise beyond resting carbohydrate oxidation [6].

### 2.6 Resting Metabolic Rate

On Days 0, 2, and 4 of both NORM and LOW trials, RMR was assessed with indirect calorimetry using a Cosmed Q‐NRG Metabolic Monitor (Cosmed Srl, Rome, Italy), calibrated as per manufacturer's directions. The assessment lasted a total of 30 min with only the final 20 min used to determine RMR [26]. RER values were used to determine resting carbohydrate and fat oxidation using non‐protein respiratory quotient values [27]. As RMR was not measured on Days 1 and 3, resting carbohydrate oxidation for these days was estimated via linear interpolation from adjacent measurements.

### 2.7 Body Composition Assessment

At baseline, and on Days 0 through to 4, participants completed a fasted body composition assessment via BIA (SECA mBCA 515; SECA GMBH, Hamburg, Germany), following manufacturer guidelines and the computer‐linked analysis software (SECA Analytics 115: SECA GMBH, Hamburg, Germany). Participants were requested to void their bladder before completing the assessment.

### 2.8 Venous Blood Sampling and Storage

Fasting venous blood samples were collected daily in blood collection tubes (EDTA, lithium heparin and serum, BD Vacutainers, Becton Dickinson U.K. Limited, Wokingham, UK). Tubes were centrifuged (3000 rpm for 10 min at 4°C) and the supernatant was stored at −80°C until later analysis.

### 2.9 Exercise Protocol

On Days 0 through to 4 participants completed a cycling session including four 3 min warm‐up stages from 50 to 125 W, with increment of 25 W every 3 min. Afterwards, participants completed a steady state (SS) cycling exercise at 95% LTP 1 until achieving 15 kcal kg FFM^−1^ of NEEE calculated from indirect calorimetry values (Moxus modular metabolic system, AEI technologies, Pittsburgh, PA, USA). Following 30 min of exercise, participants were provided with a 5 min rest period, where they dismounted the cycle ergometer (Lode Corival cpet: Lode, Groningen, the Netherlands). To avoid hypoglycemia during fasted exercise, at the 30 min rest point, participants consumed a carbohydrate gel containing 22 g CHO, 0 g PRO, 0 g FAT, 87 Kcal (SIS GO, Science in Sport, Nelson, UK). At the end of each warm‐up stage and SS exercise, heart rate, RPE, capillary blood glucose and lactate were recorded. While total exercise time was recorded once the aimed NEEE was achieved. On Day 4, only the first 30 min of the exercise session was performed, which included 12 min of warm‐up and 18 min at LTP1.

### 2.10 Skeletal Muscle Biopsy

A skeletal muscle biopsy was obtained from the lateral portion of the vastus lateralis muscle on Day 0 and Day 4 of NORM and LOW using a 5 mm Weil‐Blakesley conchotome after administering a local anesthetic (0.5% Marcaine, Bupivacaine hydrochloride, Kays Medical, Liverpool, UK). A single muscle biopsy was collected at each day on a separate incision site.

### 2.11 Venous Blood Analyses

Plasma glucose, β‐hydroxybutyrate (βHB), non‐esterified free fatty acids (NEFA), and glycerol concentrations were analyzed using commercially available kits using the RX Daytona+ (Randox Laboratories Ltd., Crumlin, UK: assay codes GL8319, FA115, GY105, and RB1007 respectively). Commercially available enzyme‐linked immunosorbent assay (ELISA) was used to measure plasma erythropoietin (EPO; Abcam, Cambridge, UK), total triiodothyronine (T_3_; DiaMetra S.r.l, Perugia, Italy), growth/differentiation factor‐15 and hepcidin (GDF‐15; R&D Systems, Minneapolis, MN, USA), leptin and serum insulin‐like growth factor 1 (IGF‐1; for both, DRG International Inc., Springfield, NJ, USA). While serum insulin, serum total testosterone, ß‐CTX, and P1NP concentrations were analyzed by a commercial laboratory (Liverpool Clinical Laboratories, Liverpool, UK) using electrochemiluminescence immunoassay (*ECLIA*) on a Roche Cobas e801 analyzer with Roche assay kits (10 059 for insulin, 10 020 for testosterone, 10 062 for ß‐CTX, 10 119 for P1NP) and calibrators (Roche Diagnostics Ltd., West Sussex, UK). Plasma metabolites intra‐assay coefficient of variation (CV) was 1.3% ± 1.0%, 2.9% ± 3.2%, 0.4% ± 0.4%, and 1.7% ± 3.0% for glucose, βHB, NEFA, glycerol, respectively. For EPO, total T_3_, GDF‐15, leptin, IGF‐1, and hepcidin the intra‐assay CV was 4.6% ± 3.2%, 6.3% ± 4.3%, 1.0% ± 1.0%, 6.8% ± 5.2%, 4.0% ± 3.3%, 5.3% ± 3.3%, respectively.

### 2.12 Muscle Glycogen Assay

Muscle glycogen concentration was determined using the acid hydrolysis method utilizing freeze‐dried muscle samples [28, 29]. Glucose concentration was quantified with the hexokinase method using a commercially available kit (GL8319; Randox Laboratories, Crumlin, UK), and glycogen concentration (mmol kg^−1^ dry mass) was then calculated. Samples were analyzed in duplicate and intra‐assay CV was 8.7% ± 10.6%.

### 2.13 Skeletal Muscle Proteomics

#### 2.13.1 Preparation of Muscle for Proteomic Analysis

Muscle samples were ground in liquid nitrogen a Cellcrusher (Cellcrusher, Cork, Ireland), then homogenized on ice in 10 volumes of 1% Triton X‐100, 50 mM Tris, pH 7.4 (including complete protease inhibitor; Roche Diagnostics, Lewes, United Kingdom) using a PolyTron homogenizer (KINEMATICA, PT 1200 E) followed by sonication (Fisherbrand). Homogenates were incubated on ice for 15 min, then centrifuged at 1000 × *g*, 4°C, for 5 min to fractionate myofibrillar (pellet) from soluble (supernatant) proteins. Myofibrillar proteins were resuspended in a half‐volume of homogenization buffer followed by centrifugation at 1000 × *g*, 4°C, for 5 min. The washed myofibrillar pellet was then solubilized in lysis buffer (7 M urea, 2 M thiourea, 4% CHAPS, 30 mM Tris, pH 8.5). Aliquots of protein were precipitated in 5 volumes of ice‐cold acetone and incubated overnight at −20°C and resuspended in 200 μL of lysis buffer. Proteins were cleared by centrifugation at 12 000 × *g*, 20°C, for 20 min. Total protein concentration (μg μL^−1^) was quantified against bovine serum albumin (BSA) standards using the Bradford assay (#23236; Thermo Scientific), according to the manufacturer's instructions.

Tryptic digestion was performed using the filter‐aided sample preparation (FASP) method [30]. Aliquots containing 100 μg protein were resuspended in 200 μL of UA buffer (8 M urea, 100 mM Tris, pH 8.5). Proteins were incubated at 37°C for 15 min in UA buffer containing 100 mM dithiothreitol followed by incubation (20 min at 4°C) protected from light in UA buffer containing 50 mM iodoacetamide. UA buffer was exchanged for 50 mM ammonium bicarbonate and sequencing‐grade trypsin (Promega V5113, Madison, WI, USA) was added at an enzyme to protein ratio of 1:50. Digestion was allowed to proceed at 37°C overnight then peptides were collected in 100 μL of 50 mM ammonium bicarbonate containing 0.2% (v/v) trifluoroacetic acid. Samples containing 4 μg of peptides were de‐salted using C_18_ Zip‐tips (Millipore) and eluted in 40% acetonitrile and 0.1% trifluoroacetic acid. Peptide solutions were dried by vacuum centrifugation and peptides were resuspended in 20 μL of loading buffer (2% (v/v) acetonitrile, 0.1% (v/v) formic acid) containing 10 fmol μL yeast ADH1 (MassPrep standard, 186 002 328; Waters Corp., Milford, MA) in preparation for LC–MS/MS analysis.

#### 2.13.2 Liquid Chromatography‐Tandem Mass Spectrometry

Data‐dependent analysis of digests from myofibrillar and soluble protein fractions was performed using an Ultimate 3000 RSLCTM nano system (Thermo Scientific) coupled to a Q‐Exactive orbitrap mass spectrometer (Thermo Scientific). Samples (myofibrillar fraction: 3 μL corresponding to 600 ng of peptides, soluble fraction: 2 μL corresponding to 400 ng of peptides) were loaded onto the trapping column (Thermo Scientific, PepMap NEO, 5 μm C18, 300 μm × 5 mm), using ulPickUp injection, for 1 min at a flow rate of 25 μL min^−1^ with 2% (v/v) acetonitrile and 0.1% (v/v) formic acid. Samples from myofibrillar fraction were resolved on a 150 mm analytical column (Easy‐Spray C_18_ 75 μm, 3 μm column) using a gradient of 97.5% A (0.1% formic acid) 2.5% B (79.9% ACN, 20% water, 0.1% formic acid) to 55% A 45% B over 31 min at a flow rate of 300 nL min^−1^. Samples from soluble fraction were resolved on a 500 mm analytical column (Easy‐Spray C18 75 μm, 2 μm column) using a gradient of 97.5% A (0.1% formic acid) 2.5% B (79.9% ACN, 20% water, 0.1% formic acid) to 50% A 50% B over 60 min at a flow rate of 300 nL min^−1^. Data acquisition included MS1 scans at 70 000 resolution at *m/z* 200 (AGC set to 3^e6^ ions with a maximum fill time of 240 ms) and data‐dependent selection of the top‐10 precursors selected from a mass range of *m/z* 395–1580 (myofibrillar fraction) or *m/z* 300–1600 (soluble fraction). MS2 data were acquired after HCD fragmentation (normalized collision energy 30) in the orbitrap analyzer at 17 500 resolution at *m/z* 200 (AGC target 5e^4^ ions with a maximum fill time of 80 ms) and a 30 s dynamic exclusion window was used to avoid repeated selection of peptides for MS/MS analysis.

#### 2.13.3 Label‐Free Quantitation of Protein Abundance

Progenesis Quantitative Informatics for Proteomics (QI‐P; Nonlinear Dynamics, Waters Corp., Newcastle, UK, Version 4.2) was used for label‐free quantitation consistent with our previous work [16, 31, 32]. Prominent ion features were used as vectors to warp each data set to a common reference chromatogram. An analysis window of 7–35 min and 395–1580 *m/z* (myofibrillar fraction) or 10–130 min and 300–1600 *m/z* (soluble fraction) were selected. Log‐transformed MS data were normalized by inter‐sample abundance ratio, and relative protein abundances were calculated using nonconflicting peptides only. Abundance data were then normalized to the 3 most abundant peptides of yeast ADH1 to derive abundance measurements in fmol μg^−1^ protein [33]. MS/MS spectra were exported in Mascot generic format and searched against the Swiss‐Prot database restricted to Homo‐sapiens (20 272 sequences) using a locally implemented Mascot server (v.2.8; www.matrixscience.com). The enzyme specificity was trypsin with 2 allowed missed cleavages, carbamidomethylation of cysteine (fixed modification) and oxidation of methionine (variable modification). *M/Z* error tolerances of 10 ppm for peptide ions and 20 ppm for fragment ion spectra were used. Peptide results were filtered to 1% FDR based on decoy search and at least one unique peptide was required to identify each protein. The Mascot output (xml format), restricted to non‐homologous protein identifications was recombined with MS profile data in Progenesis.

### 2.14 Statistical Analyses

A two‐way repeated measures analysis of variance (RM‐ANOVA, 2 × 5) was performed to analyze all body composition data, blood metabolites, hormones, bone/collagen markers, and all exercise metrics, while a RM‐ANOVA (2 × 3) analyzed RMR parameters. Normality was tested using the Shapiro–Wilk test, and results were expressed as mean ± standard deviation. Sphericity was tested using Mauchly's test, and Greenhouse–Geisser correction was applied if violated. Due to missing data points a general linear mixed modeling (LMM) was used to analyze skeletal muscle glycogen concentrations. Models included fixed effect for time (Days) and treatment (NORM or LOW) with random intercepts for subjects. Statistical significance of the fixed effect was determined using Type III tests with Satterthwaite approximation degrees of freedom. If significant main effects or interactions effects were observed, *post hoc* testing was used with Bonferroni's correction, with multiplicity‐adjusted *p* values reported when comparing NORM to LOW at respective time points. Significance was considered at *p* ≤ 0.05, effect size measures were reported for significant main effects as partial eta squared (${ƞ}_{p}^{2}$: small effect ≤ 0.05; moderate effect = 0.05–0.14; large effect ≥ 0.14) [34] for RM‐ANOVA and semi‐partial *r* ^*2*^ [35] for LMM (*r* ^*2*^: small effect ≤ 0.02; moderate effect = 0.02–0.26; large effect ≥ 0.26) [36]. A paired *t*‐test was used to determine differences in absolute and relative energy and macronutrient intake. Sample size was determined based on a priori power analysis (endocrine parameters) and resource constraints (proteomics) [37] based on the effect size of and variability of testosterone in response to low energy availability in males [38] with 8 participants required for α error probability of 0.05, and an 1‐*β* error probability of > 0.9. Analyses were performed in SPSS v30 (IBM Corp, Armonk, NY, USA), and figures were designed using GraphPad Prism v10 (GraphPad Software, San Diego, CA). Skeletal muscle proteomic data was analyzed using R (Version 4.2.2). Within‐subject 2‐factor ANOVA of time (Day 0 versus Day 4) and treatment (NORM versus LOW) was used to determine interaction. Statistical significance was set at *p* < 0.05. A nominal *p*‐value threshold without applying multiple‐testing correction elevates false‐positive risk and data should be interpreted as a hypothesis‐generating resource. Protein–protein interaction analysis was performed using the STRING database (v12.0) [39].

## 3 Results

### 3.1 Exercise Energy Expenditure and Dietary Intake

From Day 0 to Day 3, participants' average absolute and relative EI were not different between treatments. Daily mean absolute and relative EI were 3927 ± 460 kcal and 60 ± 0 kcal kg FFM^−1^ for NORM, and 3924 ± 458 kcal and 60 ± 0 kcal kg FFM^−1^ for LOW (for both, *p* > 0.05) (Table 1). Relative NEEE was not different between treatments (NORM: 15 ± 0 kcal kg FFM^−1^; LOW: 15 ± 0 kcal kg FFM^−1^; *p* > 0.05). There were no significant (for all, *p* > 0.05) treatment or interaction effects for total exercise time (NORM: 89 ± 12 min; LOW: 87 ± 13 min), % V̇O_2max_ (NORM: 63% ± 4%; LOW: 63% ± 4%) and power output during SS exercise (NORM: 152 ± 37 W; LOW: 152 ± 37 W), absolute NEEE (NORM: 982 ± 3 kcal; LOW: 983 ± 3 kcal), and EA (NORM: 45 ± 0 kcal kg FFM^−1^; LOW: 45 ± 0 kcal kg FFM^−1^).

### 3.2 Body Composition

Body mass (BM) showed a main effect of treatment (*p* = 0.011, ${ƞ}_{p}^{2}$ = 0.63—large), and an interaction (*p* < 0.001, ${ƞ}_{p}^{2}$ = 0.72—large), decreasing 1.1 ± 0.9 kg in LOW (*p* = 0.011) on Day 4 compared to Day 0, but showing no significant changes within NORM (Table 2). There was no interaction or main effect for treatment and time for fat mass (FM, *p* > 0.05). There was a main effect of treatment, time, and interaction for fat‐free mass (FFM) and total body water (TBW, *p* < 0.05, ${ƞ}_{p}^{2}$ > 0.33—large, for all). BM, TBW and FFM were higher for NORM versus LOW from Day 2 onwards (*p* < 0.05 at all time points).

**TABLE 2:** Body composition data for NORM and LOW.

	Day 0	Day 1	Day 2	Day 3	Day 4	*p*
BM (kg)						
NORM	78.8 ± 11.3	79.5 ± 11.5	79.4 ± 11.5*	79.6 ± 11.5***	79.3 ± 11.4***	**Tr, *p* = 0.011** T, *p* = 0.098 **Tr × T, *p* < 0.001**
LOW	79.2 ± 11.6^ab^	79.0 ± 11.3^a^	78.4 ± 11.3^a^	78.5 ± 11.4^ab^	78.1 ± 11.2^b^	
FM (kg)						
NORM	13.4 ± 4.7	12.9 ± 4.7	13.3 ± 4.7	12.9 ± 4.7	13.3 ± 4.5	Tr, *p* = 0.230 T, *p* = 0.216 Tr × T, *p* = 0.214
LOW	13.7 ± 4.9	13.4 ± 4.8	13.5 ± 4.7	13.6 ± 4.5	13.5 ± 4.6	
FFM (kg)						
NORM	65.4 ± 7.5^ac^	66.5 ± 8.0^abc^	66.1 ± 7.8**^abc^	66.7 ± 7.9***^b^	65.8 ± 7.8*^c^	**Tr, *p* = 0.003** **T, *p* = 0.019** **Tr × T, *p* = 0.001**
LOW	65.5 ± 8.5	65.5 ± 8.1	64.9 ± 7.5	64.9 ± 7.8	64.6 ± 7.6	
TBW (L)						
NORM	47.7 ± 5.6^ac^	48.5 ± 5.9^abc^	48.1 ± 5.8**^abc^	48.7 ± 5.8***^b^	48.0 ± 5.8**^c^	**Tr, *p* = 0.003** **T, *p* = 0.020** **Tr × T, *p* < 0.001**
LOW	47.7 ± 6.2	47.8 ± 5.9	47.4 ± 5.6	47.4 ± 5.8	47.1 ± 5.6	

*Note:* Values are means ± SD (*n* = 8). Bold *p* values indicate statistically significant treatment, time, or interaction effects (*p* < 0.05). *p < 0.05, **p < 0.010, ***p < 0.001 for within time point comparison between NORM vs. LOW. Time‐points not sharing letters in superscript within each treatment are statistically different (*p* < 0.05).

Abbreviations: BM, body mass; FFM, fat‐free mass; FM, fat mass; TBW, total body water.

### 3.3 Resting Metabolic Rate

**FIGURE 2:**
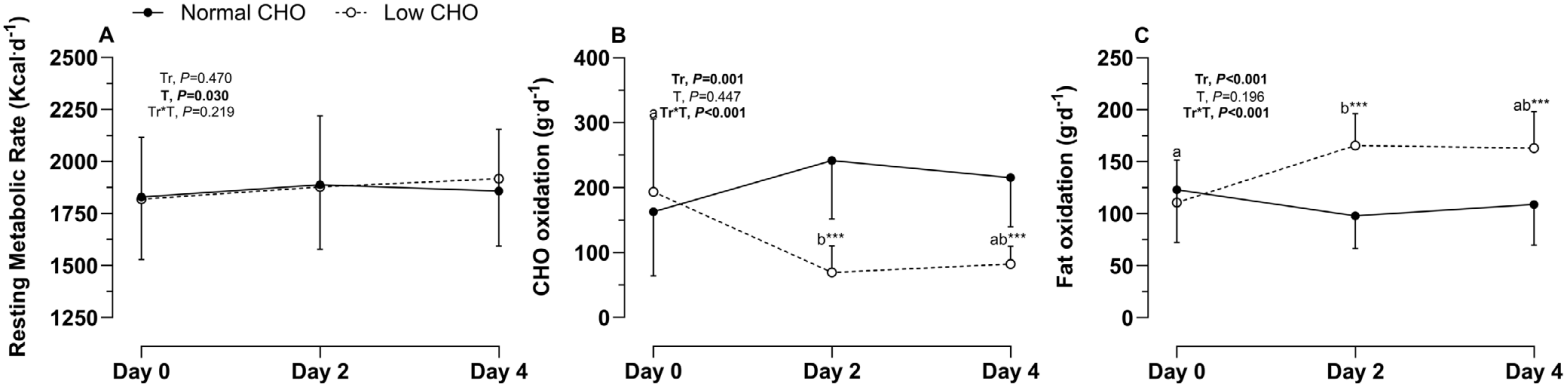
RMR (A), CHO oxidation (B) and fat oxidation (C). Data are presented as mean ± SD (*n* = 8). ***p < 0.001 for within time point comparison between NORM vs. LOW. *p* < 0.05 for time points not sharing lowercase letters in LOW for panel (B).

RMR showed a main effect on time (*p* = 0.030, ${ƞ}_{p}^{2}$ = 0.40—large), but no treatment or interaction effect (*p* > 0.05; Figure 2A). For CHO and fat oxidation there was a main effect on treatment (*p* = 0.001, ${ƞ}_{p}^{2}$ = 0.79—large; *p* < 0.001, ${ƞ}_{p}^{2}$ =0.81—large, respectively) and interaction (for both, *p* < 0.001, ${ƞ}_{p}^{2}$ = 0.69—large), but no main effect on time (for both, *p* > 0.05). CHO oxidation was significantly higher in NORM compared to LOW at Day 2 (me an difference: 172 g day^−1^, 95% CI: 110 to 235, *p* < 0.001) and Day 4 (mean difference: 133 g day^−1^, 95% CI: 80 to 186, *p* < 0.001; Figure 2B). Fat oxidation was significantly higher in LOW comparted to NORM at Day 2 (mean difference: 67 g day^−1^, 95% CI: 44 to 91, *p* < 0.001) and Day 4 (mean difference: 54 g day^−1^, 95% CI: 32 to 76, *p* < 0.001; Figure 2C).

### 3.4 Physiological and Metabolic Responses During Exercise

Physiological and metabolic responses during exercise at a range of intensities are shown in Table 4. There were no differences in blood glucose during exercise, but we observed a clear time effect in reduced circulating lactate in LOW compared to NORM. As expected, there was also a time × treatment interaction with the reduction in the oxidation of carbohydrates with a parallel increase in the oxidation of fat in LOW. Gross efficiency increased with time during SS in NORM, but not in LOW, reaching a higher efficiency in NORM compared to LOW on Days 3 and 4. Similarly, there was also a reduction of heart rate in NORM with time, but not in LOW. RPE was higher in LOW compared to NORM at all intensities above 50 W.

### 3.5 Dietary Carbohydrate Availability

There was a significant main effect of treatment (*p* < 0.001, ${ƞ}_{p}^{2}$ = 0.98—large), time (*p* < 0.001, ${ƞ}_{p}^{2}$ = 0.58—large), and interaction (*p* < 0.001, ${ƞ}_{p}^{2}$ = 0.80—large) on CHO availability (Table 3). From Day 0 to Day 3, CHO availability was greater in NORM versus LOW (for all, *p* < 0.001). Within LOW, CHO availability was significantly higher on Days 1, 2, and 3 compared to Day 0, due to a reduced reliance of carbohydrate use during exercise (Tables 3 and 4).

**TABLE 3:** Daily carbohydrate availability.

	Day 0	Day 1	Day 2	Day 3	*p*
Total exercise CHO oxidation (g day^−1^)					
NORM	171 ± 41***	179 ± 23***	188 ± 31***	183 ± 23***	**Tr,** ***p*** **<** **0.001** **T,** ***p*** **= 0.010** **Tr** **×** **T** **,** ***p* < 0.001**
LOW	181 ± 49^a^	130 ± 3^b^	120 ± 24^bc^	106 ± 20^cd^	
Daily CHO intake (g day^−1^)					
NORM	607 ± 73	607 ± 73	607 ± 73	607 ± 73	
LOW	117 ± 15	117 ± 15	117 ± 15	117 ± 15	
Daily CHO availability (g day^−1^)					
NORM	446 ± 53***	440 ± 57***	433 ± 54***	438 ± 60***	**Tr,** ***p*** **<** **0.001** **T,** ***p** **<*** **0.001** **Tr** **×** **T,** ***p* < 0.001**
LOW	−52 ± 35^a^	−6 ± 24^b^	0 ± 13^bc^	15 ± 15^cd^	

*Note:* Daily CHO availability was operationally defined as the difference of the total daily carbohydrate intake and the net amount of carbohydrates oxidized during exercise. Values are means ± SD (*n* = 8). Bold *p* values indicate statistically significant treatment, time, or interaction effects (*p* < 0.05). ***p < 0.001 for within time point comparison between NORM vs. LOW. Time points not sharing letters in superscript within each treatment are statistically different (*p* < 0.05).

**TABLE 4:** Daily exercise data for 3‐min warm‐up stages (50–125 W) and mean data during steady state (*SS*) exercise at 95% lactate turn‐point.

Parameter	Power	NORM					LOW					*p*		
		D 0	D 1	D 2	D 3	D 4	D 0	D 1	D 2	D 3	D 4	Time	Treatment	Interaction
HR (Beats min^−1^)	50 W	88 ± 10	91 ± 10	91 ± 10	91 ± 13	91 ± 9	90 ± 7	93 ± 7	94 ± 7	98 ± 10	99 ± 7	0.780	**0.033**	0.730
	75 W	98 ± 12	99 ± 10	100 ± 11	102 ± 12	98 ± 9*	99 ± 7	100 ± 10	104 ± 8	106 ± 11	107 ± 7	**0.012**	0.064	**0.030**
	100 W	111 ± 11	108 ± 10	111 ± 10	111 ± 14	108 ± 11	109 ± 8^ab^	111 ± 12^a^	114 ± 9^ab^	118 ± 12^b^	118 ± 11^ab^	**0.001**	**0.025**	**0.033**
	125 W	120 ± 13	121 ± 12	122 ± 14	121 ± 16	119 ± 12	120 ± 1	123 ± 13	125 ± 14	127 ± 14	128 ± 13	**0.024**	0.052	**0.031**
	SS	142 ± 8^a^	142 ± 10^a^	143 ± 10^a^	139 ± 9^a^	133 ± 10^b^	142 ± 11^ab^	144 ± 12^ab^	147 ± 10^a^	145 ± 9^a^	141 ± 11^b^	**< 0.001**	0.075	**0.011**
RPE (AU)	50 W	7 ± 1	7 ± 1	7 ± 1	7 ± 1	7 ± 1	7 ± 1	7 ± 1	7 ± 1	7 ± 1	7 ± 1	0.533	0.598	**0.013**
	75 W	8 ± 2	8 ± 2	8 ± 1	8 ± 2	7 ± 2*	8 ± 2	8 ± 2	8 ± 2	8 ± 2	8 ± 1	0.359	**0.026**	**0.045**
	100 W	9 ± 2	8 ± 1	8 ± 1	8 ± 2	8 ± 2	8 ± 2	9 ± 2	9 ± 2	9 ± 2	9 ± 2	0.609	**0.030**	**0.010**
	125 W	10 ± 2	9 ± 2	9 ± 2	9 ± 2	9 ± 2	9 ± 2	10 ± 3	10 ± 2	11 ± 2	10 ± 2	0.351	**0.008**	0.096
	SS	13 ± 2	12 ± 2	12 ± 2	12 ± 2	11 ± 2	12 ± 1^a^	13 ± 2^b^	13 ± 2^b^	13 ± 2^ab^	12 ± 2^ab^	**0.040**	**0.019**	**0.004**
Lactate (mmol L^−1^)	50 W	1.2 ± 0.5	1.0 ± 0.3	0.9 ± 0.2	1.3 ± 0.9	1.2 ± 0.3	1.1 ± 0.2	1.0 ± 0.4	1.1 ± 0.3	0.9 ± 0.2	0.9 ± 0.3	0.515	0.160	0.413
	75 W	1.2 ± 0.4	0.8 ± 0.3	1.0 ± 0.3	0.9 ± 0.3	0.9 ± 0.1	1.2 ± 0.3	0.9 ± 0.5	1.0 ± 0.3	0.8 ± 0.1	0.9 ± 0.3	**0.009**	0.905	0.843
	100 W	1.4 ± 0.8	1.2 ± 0.4	1.1 ± 0.3	1.1 ± 0.5	1.0 ± 0.3	1.3 ± 0.4	1.0 ± 0.5	1.1 ± 0.2	0.9 ± 0.2	0.9 ± 0.4	**0.003**	0.186	0.711
	125 W	1.9 ± 1.2	1.3 ± 0.6	1.6 ± 0.5	1.3 ± 0.5	1.3 ± 0.5	1.7 ± 0.7	1.3 ± 0.7	1.1 ± 0.4	1.1 ± 0.4	0.9 ± 0.3	**< 0.001**	0.107	0.662
	SS	2.8 ± 1.1	2.5 ± 0.6	2.4 ± 0.5	2.4 ± 0.5	1.9 ± 0.5	2.5 ± 0.7	1.7 ± 0.3	1.7 ± 0.5	1.6 ± 0.3	1.4 ± 0.3	**0.019**	**0.004**	0.296
Glucose (mmol L^−1^)	50 W	4.37 ± 0.35	4.64 ± 0.30	4.60 ± 0.24	4.33 ± 0.47	4.45 ± 0.16	4.18 ± 0.68	4.32 ± 0.41	4.33 ± 0.16	4.42 ± 0.35	4.04 ± 0.74	0.131	**0.008**	0.369
	75 W	4.43 ± 0.24	4.50 ± 0.42	4.46 ± 0.31	4.35 ± 0.55	4.30 ± 0.39	4.42 ± 034	4.21 ± 0.24	4.16 ± 0.63	4.33 ± 0.39	4.03 ± 0.75	0.271	**0.041**	0.704
	100 W	4.33 ± 0.48	4.41 ± 0.36	4.08 ± 0.66	4.14 ± 0.29	4.24 ± 0.38	4.47 ± 0.52	4.20 ± 0.27	4.25 ± 0.32	4.36 ± 0.25	4.01 ± 0.68	0.171	0.844	0.148
	125 W	4.49 ± 0.22	4.27 ± 0.39	4.27 ± 0.45	4.14 ± 0.40	4.29 ± 0.41	4.53 ± 0.33	4.17 ± 0.39	4.12 ± 0.43	4.33 ± 0.27	3.84 ± 0.68	**0.018**	0.100	0.080
	SS	4.31 ± 0.34	4.17 ± 0.35	4.09 ± 0.52	3.76 ± 0.52	4.19 ± 0.33	4.46 ± 0.27^a^	4.01 ± 0.37^b^	3.95 ± 0.43^b^	3.98 ± 0.40^b^	3.63 ± 0.53^b^	**< 0.001**	0.387	**0.031**
CHO Ox (g min^−1^)	50 W	0.29 ± 0.24	0.35 ± 0.26	0.45 ± 0.27	0.48 ± 0.22***	0.40 ± 0.14***	0.37 ± 0.30	0.10 ± 0.10	0.11 ± 0.19	0.12 ± 0.11	0.07 ± 0.08	0.371	**< 0.001**	**0.003**
	75 W	0.88 ± 0.39	0.88 ± 0.17*	0.91 ± 0.33*	0.98 ± 0.25***	0.96 ± 0.08***	0.84 ± 0.36^a^	0.57 ± 0.26^ab^	0.43 ± 0.26^ab^	0.36 ± 0.20^ab^	0.29 ± 0.19^b^	0.129	**< 0.001**	**< 0.001**
	100 W	1.32 ± 0.56	1.38 ± 0.33*	1.40 ± 0.34**	1.44 ± 0.37***	1.40 ± 0.15***	1.31 ± 0.43^a^	1.02 ± 0.31^a^	0.83 ± 0.24^ab^	0.69 ± 0.25^b^	0.51 ± 0.26^b^	**0.017**	**< 0.001**	**< 0.001**
	125 W	1.81 ± 0.67	1.92 ± 0.40***	1.97 ± 0.41***	1.96 ± 0.39*	1.94 ± 0.21***	1.87 ± 0.54^a^	1.41 ± 0.49^b^	1.20 ± 0.36^abc^	1.00 ± 0.58^abc^	0.83 ± 0.33^c^	**0.032**	**< 0.001**	**0.003**
	SS	2.08 ± 0.43^ab^	2.24 ± 0.37***^ab^	2.29 ± 0.47***^ab^	2.19 ± 0.47***^a^	2.47 ± 0.59***^b^	2.18 ± 0.56^a^	1.64 ± 0.38^b^	1.54 ± 0.32^b^	1.34 ± 0.29^b^	1.36 ± 0.27^b^	**0.011**	**< 0.001**	**< 0.001**
Fat Ox (g min^−1^)	50 W	0.51 ± 0.12	0.48 ± 0.12*	0.44 ± 0.11*	0.42 ± 0.09***	0.47 ± 0.14*	0.51 ± 0.15	0.60 ± 0.06	0.65 ± 0.11	0.61 ± 0.08	0.64 ± 0.15	0.578	**< 0.001**	**0.003**
	75 W	0.41 ± 0.15	0.39 ± 0.07*	0.36 ± 0.14***	0.34 ± 0.11***	0.37 ± 0.10***	0.41 ± 0.15^a^	0.53 ± 0.13^ab^	0.61 ± 0.12^ab^	0.63 ± 0.11^ab^	0.65 ± 0.16^b^	0.056	**< 0.001**	**< 0.001**
	100 W	0.37 ± 0.22	0.35 ± 0.13***	0.31 ± 0.14***	0.29 ± 0.13***	0.33 ± 0.15***	0.37 ± 0.21^a^	0.52 ± 0.13^a^	0.60 ± 0.12^ab^	0.68 ± 0.14^b^	0.69 ± 0.19^b^	**0.028**	**< 0.001**	**< 0.001**
	125 W	0.34 ± 0.25^a^	0.27 ± 0.16***^ab^	0.25 ± 0.18***^b^	0.24 ± 0.14*^ab^	0.26 ± 0.14***^ab^	0.30 ± 0.24^a^	0.52 ± 0.21^b^	0.61 ± 0.15^abc^	0.70 ± 0.25^bc^	0.71 ± 0.19^c^	**0.018**	**< 0.001**	**< 0.001**
	SS	0.49 ± 0.20^abc^	0.44 ± 0.12***^b^	0.39 ± 0.11***^abc^	0.42 ± 0.10***^b^	0.26 ± 0.09***^c^	0.43 ± 0.22^a^	0.70 ± 0.22^b^	0.74 ± 0.14^b^	0.83 ± 0.16^b^	0.79 ± 0.20^b^	**< 0.001**	**< 0.001**	**< 0.001**
GE (%)	50 W	11.5 ± 1.2	11.9 ± 1.4	11.9 ± 0.9	12.0 ± 0.9	11.9 ± 1.7	11.3 ± 1.5	11.8 ± 1.2	11.3 ± 1.2	11.4 ± 0.9	11.5 ± 1.3	0.482	**< 0.001**	0.908
	75 W	14.3 ± 1.0	14.6 ± 1.2	15.0 ± 1.1	14.9 ± 0.9	14.6 ± 1.8	14.5 ± 1.2	14.4 ± 1.4	14.1 ± 1.1	14.3 ± 1.0	13.7 ± 1.3	0.515	0.071	0.671
	100 W	16.1 ± 1.1	16.1 ± 1.3	16.5 ± 1.1	16.5 ± 1.2	16.3 ± 2.0	16.1 ± 1.1	15.6 ± 1.2	15.6 ± 1.1	15.3 ± 1.1	15.5 ± 1.1	0.806	**0.032**	0.710
	125 W	17.0 ± 1.0	17.2 ± 1.1	17.3 ± 1.2	17.4 ± 0.9	17.4 ± 1.6	17.1 ± 0.7	16.7 ± 0.8	16.5 ± 1.0	16.6 ± 1.0	16.7 ± 0.9	0.996	0.084	0.726
	SS	16.4 ± 1.8^a^	16.3 ± 1.9^ab^	16.6 ± 1.8^ab^	16.7 ± 1.6*^ab^	17.4 ± 1.9*^b^	16.6 ± 1.5	16.1 ± 1.7	16.1 ± 1.6	16.0 ± 1.6	16.6 ± 1.7	**0.010**	**0.030**	**0.036**

*Note:* Exercise parameters during NORM and LOW at 50 W, 75 W, 100 W, 125 W, and mean values during steady state exercise at 95% lactate turn‐point 1 (SS). Day 4 SS exercise was terminated at 30 min. Bold *p* values indicate statistically significant treatment, time, or interaction effects (*p* < 0.05). Values are means ± SD (*n* = 8). *p < 0.05, **p < 0.010, ***p < 0.001 for within time point comparisons between NORM and LOW. *p* < 0.05 for time points not sharing lowercase letters within either NORM or LOW.

Abbreviations: AU, arbitrary units; CHO Ox, carbohydrate oxidation rate; FAT Ox, fat oxidation rate; GE, gross efficiency; HR, heart rate; RPE, rating of perceived exertion.

### 3.6 Fasting Plasma Metabolites

**FIGURE 3:**
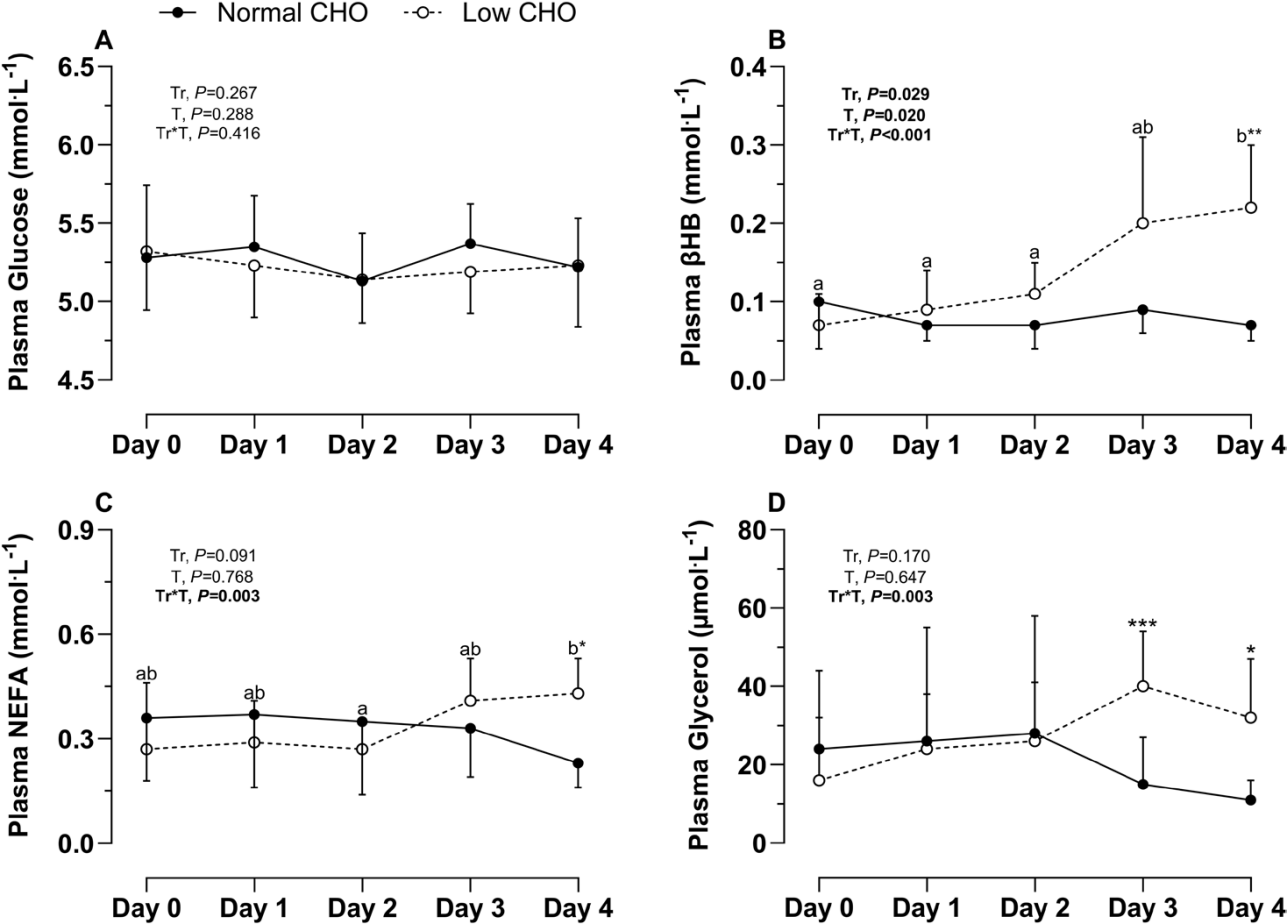
Plasma glucose (A), βHB (B), NEFA (C), glycerol (D) throughout NORM and LOW. Data are presented as mean ± SD (*n* = 8). *p < 0.05, **p < 0.010, ***p < 0.001 for within time point comparison between NORM vs. LOW. *p* < 0.05 for time points not sharing lowercase letters in LOW for panels (B, C). *βHB*, β‐hydroxybutyrate; *NEFA*, non‐esterified fatty acids.

There were no significant main effects of treatment, time, or interaction on plasma glucose concentrations (*p* > 0.05; Figure 3A). Plasma βHB concentration (Figure 3B) showed a main effect of treatment (*p* = 0.029, ${ƞ}_{p}^{2}$ = 0.52—large), time (*p* < 0.020, ${ƞ}_{p}^{2}$ = 0.46—large), and interaction (*p* < 0.001, ${ƞ}_{p}^{2}$ = 0.70—large). Only on Day 4, LOW βHB concentrations (0.22 ± 0.08 mmol L^−1^) reached levels significantly higher than Day 0 (0.07 ± 0.04, *p* = 0.046) and compared to NORM on the same day (0.07 ± 0.02 mmol L^−1^: mean difference: 0.16 mmol L^−1^, 95% CI: 0.09 to 0.22, *p* = 0.005).

For plasma NEFA and glycerol there was no main effect of treatment or time (*p* > 0.05), but there was an interaction (*p* = 0.003, ${ƞ}_{p}^{2}$ = 0.42—large) (Figure 3C,D). Plasma NEFA concentrations were significantly greater in LOW compared with NORM on Day 4 (mean difference: 0.20 mmol L^−1^, 95% CI: 0.09 to 0.32, *p* < 0.05). Similarly, plasma glycerol concentrations were significantly greater in LOW versus NORM on Days 3 (mean difference: 25 μmol L^−1^, 95% CI: 16 to 34, *p* < 0.001) and 4 (mean difference: 21 μmol L^−1^, 95% CI: 7 to 35, *p* = 0.045).

### 3.7 Endocrine Responses

**FIGURE 4:**
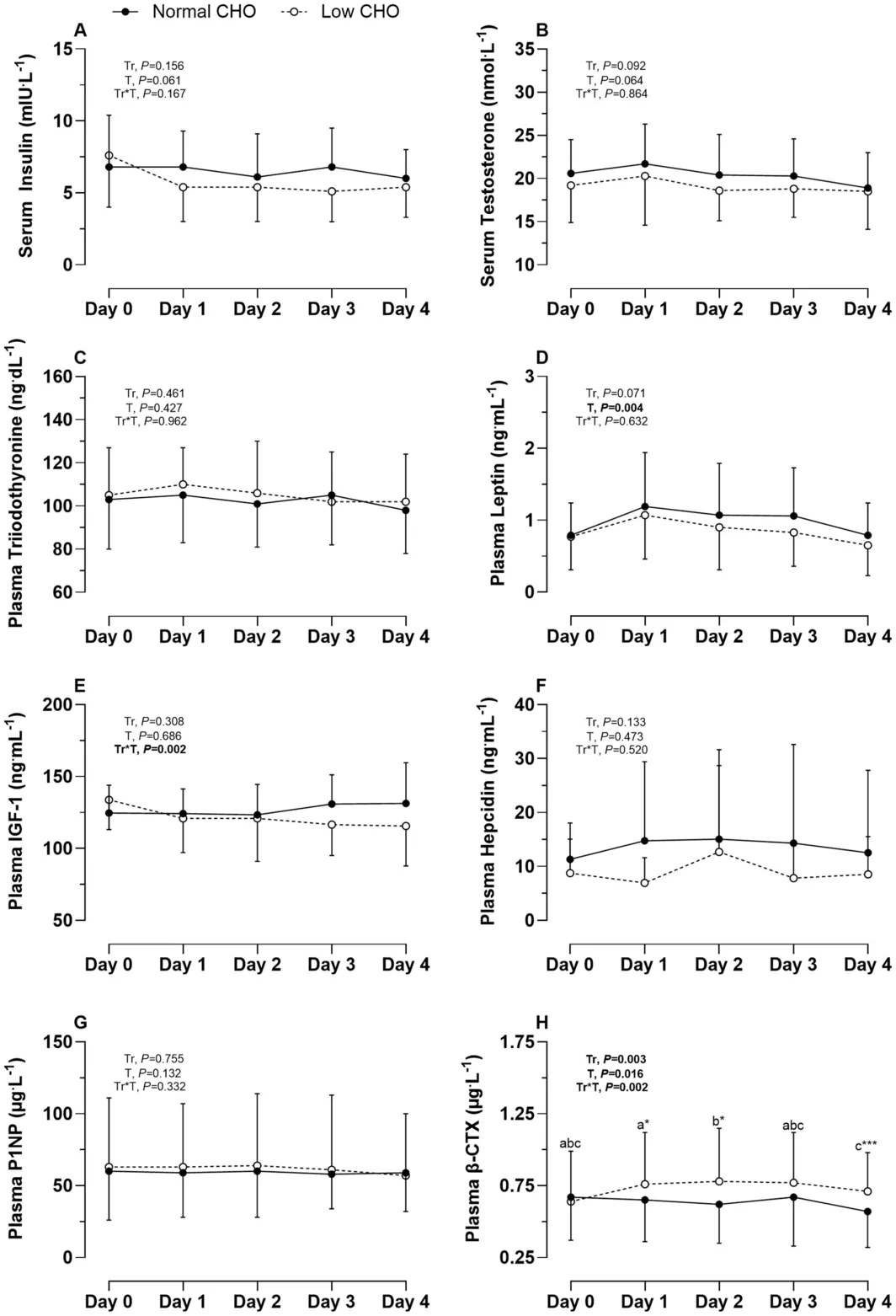
Serum insulin (A), serum testosterone (B), Plasma T3 (C), plasma leptin (D), plasma IGF‐1 (E), plasma hepcidin (F), P1NP (G), and β‐CTX (H) throughout NORM and LOW. Data are presented as mean ± SD (*n* = 8). *p < 0.05, ***p < 0.001 for within time point comparison between NORM vs. LOW. *p* < 0.05 for time points not sharing lowercase letters in LOW for panel (H) *GDF‐15*, growth differentiation factor 15; *P1NP*, procollagen type 1 n‐terminal propeptide; *β‐CTX*, β‐isomerized carboxy‐terminal cross‐linking telopeptides.

There was no significant main effect of treatment, time, or interaction on serum insulin, serum testosterone, plasma T3, plasma hepcidin, or plasma EPO concentrations (*p* > 0.05; Figure 4A–C,F). There was a main effect of time (*p* = 0.004, ${ƞ}_{p}^{2}$ =0.42—large), but no main effect on treatment or interaction for plasma leptin (*p* > 0.05; Figure 4D). Plasma IGF‐1 concentrations showed no main effect on treatment or time (*p* > 0.05) but an interaction effect (*p* = 0.002, ${ƞ}_{p}^{2}$ = 0.44—large; Figure 4E). Plasma GDF‐15 presented a main effect on treatment (*p* = 0.003, ${ƞ}_{p}^{2}$ = 0.73—large) and interaction (*p* = 0.049, ${ƞ}_{p}^{2}$ = 0.28—large), but no main effect on time (*p* > 0.05). GDF‐15 concentrations were significantly higher in NORM (362 pg mL^−1^) versus LOW (341 pg mL^−1^ *; p* = 0.003), but there was no significant differences within timepoints and between NORM and LOW for plasma IGF‐1 and GDF‐15 (for all, *p* > 0.05).

### 3.8 Markers of Bone/Collagen Formation and Resorption

There were no main effects of treatment, time or interaction for plasma P1NP (*p* > 0.05; Figure 4G). Plasma β‐CTX showed a main effect of treatment (*p* = 0.003, ${ƞ}_{p}^{2}$ = 0.73—large), time (*p* = 0.016, ${ƞ}_{p}^{2}$ = 0.42—large), and interaction (*p* = 0.002, ${ƞ}_{p}^{2}$ = 0.44—large), with significant greater concentrations on LOW compared to NORM at Day 1 (mean difference: 11 μg L^−1^, 95% CI: 4 to 18, *p* = 0.040), Day 2 (mean difference: 16 μg L^−1^, 95% CI: 7 to 24, *p* = 0.020), and Day 4 (mean difference: 14 μg L^−1^, 95% CI: 9 to 19, *p* < 0.001; Figure 4H).

### 3.9 Skeletal Muscle

#### 3.9.1 Muscle Glycogen

**FIGURE 5:**
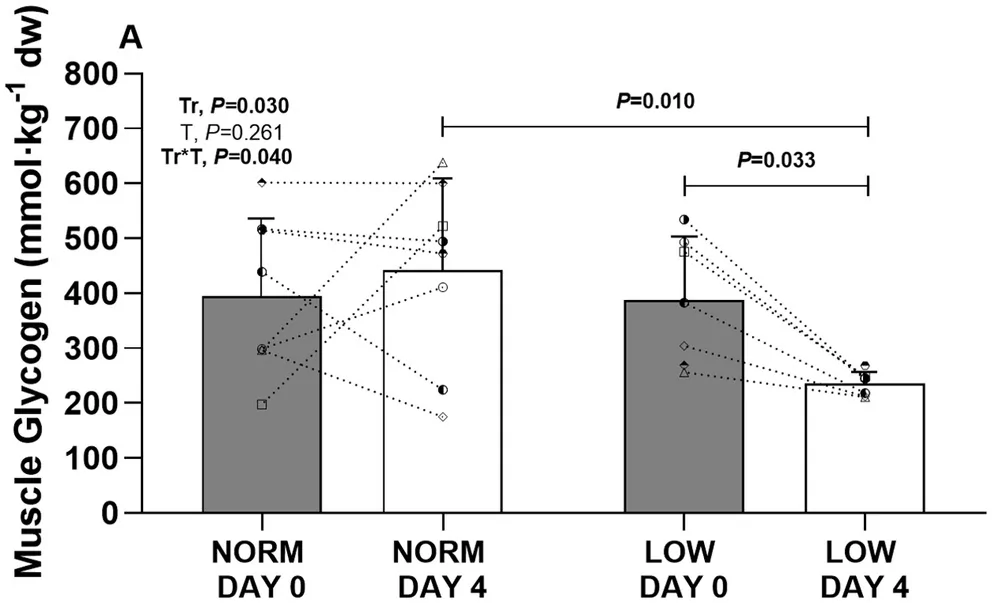
Muscle glycogen concentration before and after 4 days of NORM and LOW. Data are presented as mean ± SD (*n* = 8). A unique symbol is used to show values of each individual.

Skeletal muscle glycogen concentrations showed a main effect of treatment (*p* = 0.030, *r* ^*2*^ = 0.22—moderate) and an interaction (*p* = 0.040, *r* ^*2*^ = 0.20—moderate), but no main effect of time (*p* > 0.05; Figure 5A). Skeletal muscle glycogen concentrations remained unchanged in NORM on Day 4 (442 ± 166 mmol kg^−1^) compared to Day 0 (395 ± 142 mmol kg^−1^, *p* = 0.467), but were lower in LOW on Day 4 (236 ± 22 mmol kg^−1^), compared to LOW Day 0 (388 ± 115 mmol kg^−1^, *p* = 0.033), and within Day 4, glycogen concentrations were also lower in LOW compared to NORM (mean difference: −130 mmol kg^−1^, 95% CI: −236 to −25, *p* = 0.010), which is within predicted values [40].

#### 3.9.2 Muscle Proteomics

**FIGURE 6:**
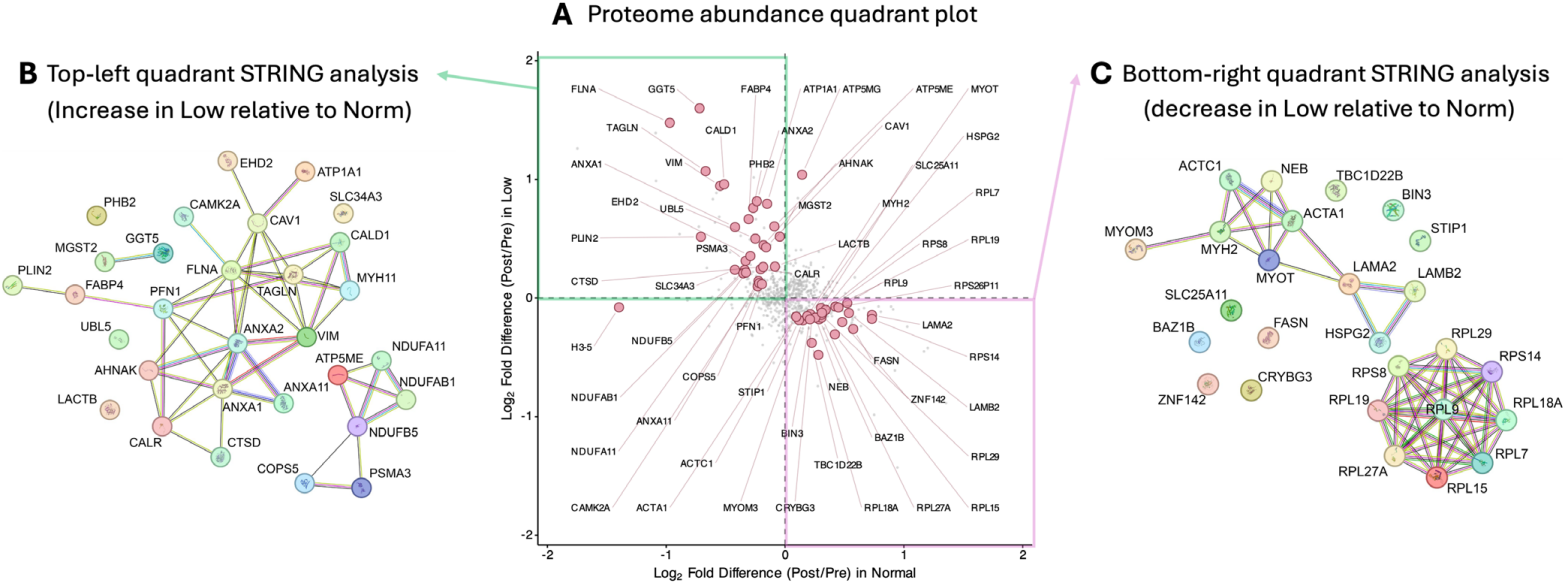
Discordant muscle proteome responses to exercise between normal and low levels of carbohydrate availability. Scatter plot of the average within‐subject log_2_ fold‐change in protein abundance between pre‐intervention (Day 0) and post‐intervention (Day 4) time points during either NORM (*x*‐axis) or LOW (*y*‐axis) (A). Data from 671 proteins with no missing values across *n* = 8 participants were analyzed using a within‐subject two‐factor ANOVA. Proteins exhibiting significant (*p* < 0.05) interaction effects between treatment (LOW vs. NORM) and time (Day 0 vs. Day 4) are highlighted in pink and are annotated with their gene name. Protein network drawn from proteins that exhibited a significant interaction effect and occupied the top‐left quadrant of the scatter plot, which represents proteins that increased in abundance during LOW but decreased in abundance NORM (B). Protein network drawn from proteins that exhibited a significant interaction effect and occupied the bottom‐right quadrant of the scatter plot, which represents proteins that decreased in abundance during LOW but increased in abundance during NORM (C). Protein interaction networks were created using the Search Tool for the retrieval of interacting genes/proteins (STRING).

Proteomic analysis was conducted on 32 muscle samples taken at 4 time points (*n* = 8 participants) prior to and after the two experimental periods (Figure 1). Stringent filtering was used to ensure robust statistical analysis of the within‐subject repeated measures experimental design. Overall, 702 proteins were confidently (FDR < 1%, > 1 unique peptide) identified, and after summing protein abundance from soluble and myofibrillar fractions and excluding proteins that were not detected in each of the four samples, 671 proteins had complete within‐subject data and were used for statistical analysis (Figure 6A).

Within‐subject 2‐factor analysis of variance highlighted 57 proteins that exhibited statistically significant (for all, *p* < 0.05) interactions between condition (low vs. normal‐carbohydrate) and time (baseline vs. post‐intervention). Thirty of the 57 significant proteins became more abundant during the LOW condition and less abundant during the NORM condition (Figure 6B). Conversely, 27 of the significant proteins exhibited the opposite response and became less abundant during the LOW condition and increased in abundance during the NORM condition (Figure 6C). See Table S1 for the complete protein list and and Figures S1 and S2 for a boxplot representation of all proteins with significant interaction effect.

The Gene Ontology (GOCC:0030659) “cytoplasmic vesicle membrane” was enriched (FDR 0.0391) amongst the proteins that increased specifically during the LOW condition, and two protein clusters were highlighted by STRING network analysis. The first cluster, composed of 19 proteins (PLIN2, FABP4, PFN1, FLNA, ANXA2, VIM, ANXA1, AHNAK, CALR, ANXA11, CTSD, TAGLN, CAV1, EHD2, CAMK2A, SLC34A3, CALD1, ATP1A1, MYH11) represents collectively proteins linked to lipid droplet coating, fatty acid uptake, vesicle transport and calcium signaling, suggesting modulation of lipid metabolism and transport. The second cluster, composed of 6 proteins shows 4 closely interlinked mitochondrial proteins corresponding to ATP synthase protein (ATPM5ME) and proteins of complex I of the electron transport chain (NDUFAB1, NDUFA11 and NDUFB5) (Figure 6B).

The bottom‐right quadrant includes 27 proteins showing an interaction (Figure 6C), which represent those that were lower in LOW relative to NORM (for all, *p* < 0.05). Gene ontology analysis identified enrichment in the biological process of translation (GO:0006412; FDR 6.32 e−05) with 9 ribosomal protein units identified (RPL27A, RPL15, RPL29, RPL18A, RPL19, RPS8, RPL7, RPL9 and RPS14), association which was also evident as a cluster in STRING analysis (Figure 6C). A second cluster was also identified with STRING analysis, which was composed of 9 proteins represented primarily by sarcomeric (MYH2, ACTA1, ACTC, MYOM3, NEB and MYOT) and extracellular proteins (LAMA2, LAMB2 and HSPG2).

## 4 Discussion

The principal finding of this study is that short‐term LCA, induced through an energy‐balanced low‐carbohydrate high‐fat diet, produced a minimal endocrine perturbation and did not reproduce the broad endocrine profile and skeletal muscle responses previously evidenced with LEA. Despite marked metabolic adaptation—including reduced muscle glycogen, elevated circulating free fatty acids, glycerol, and ketone bodies, and increased fat oxidation at rest and during exercise—concentrations of triiodothyronine, leptin, testosterone, insulin, hepcidin, and P1NP remained unchanged with LCA. In contrast, β‐CTX increased and IGF‐1 declined relative to the control condition, suggesting that bone turnover and anabolic signaling may be selectively sensitive to carbohydrate restriction independent of energy deficit. Proteomic analyses further identified a coordinated skeletal muscle phenotype characterized by enrichment of proteins involved in lipid metabolism and transport, alongside reductions in ribosomal, sarcomeric, and extracellular matrix‐associated proteins following a low‐carbohydrate high‐fat diet. However, there was no clear widespread upregulation of mitochondrial proteins as hypothesized. Collectively, these findings indicate that isolated LCA exerts limited systemic endocrine effects under energy‐balanced conditions but may promote early tissue‐specific anti‐anabolic adaptations, suggesting that acute LEA may be necessary with concomitant LCA to maximize the adaptive signal in muscle to shift it towards an oxidative phenotype, which could have important implications for carbohydrate and energy periodisation.

To our knowledge, this is the first controlled study to isolate carbohydrate availability while comprehensively assessing endocrine, metabolic, bone, iron, and skeletal muscle proteomic responses in exercising males under maintained energy availability. Previous investigations manipulating carbohydrate availability have largely focused on isolated outcomes such as bone turnover [9, 10, 12] or iron regulation [11, 12, 14], frequently in race walkers, female cohorts, or under less controlled field settings, and tightly controlled studies with skeletal muscle analyses have typically been limited to intracellular signaling and gene expression after < 24 h treatment [10, 17, 20]. By contrast, the present study integrates repeated endocrine measurements with skeletal muscle proteomics under tightly controlled dietary and exercise conditions over 4 days, allowing clearer distinction between responses attributable to carbohydrate restriction and those secondary to reduced energy availability.

The dietary intervention successfully induced the expected metabolic phenotype of LCA without altering fat mass, confirming effective substrate manipulation in the absence of energy deficit (Table 3). The increase in circulating NEFA, glycerol, and ketones (Figure 3), together with elevated fat oxidation at rest (Figure 2) and during exercise (Table 4), reduced exercise glucose concentrations (Table 4), and lower skeletal muscle glycogen (Figure 5), implies a robust shift towards lipid‐supported metabolism. However, this metabolic reorganization occurred without perturbation of endocrine markers typically linked to LEA. Due to the brain's reliance on glucose, carbohydrate restriction has been proposed as a potential driver of endocrine disruption during low energy availability [6], yet the present findings indicate that carbohydrate restriction alone is insufficient to disrupt the hypothalamic–pituitary–thyroid and hypothalamic–pituitary–gonadal axes when total energy availability is preserved over a 4‐day period. Although reductions in T3 are consistently observed during experimentally induced low energy availability in females [1] and males [16], and carbohydrate availability has been proposed to contribute to these responses [8], T3 remained unchanged in the present study. Similarly, testosterone was unaffected, consistent with experimental evidence indicating that testosterone suppression in males is not a universal feature of short‐term low energy availability [1, 5, 16]. These findings support the interpretation that endocrine suppression during LEA may be driven primarily by energetic deficit rather than carbohydrate restriction per se.

The response of bone and collagen turnover markers appears more nuanced. The isolated increase in β‐CTX, despite unchanged P1NP (Figure 4), suggests that bone resorption may be more sensitive than bone formation to acute carbohydrate restriction in this context. This aligns with previous reports showing that LCA consistently alters bone turnover markers. Unlike other studies, we did not show reductions in P1NP [9, 10, 12] but our findings align with reports of elevations in CTX with LCA [10, 12]. The present findings extend this literature by demonstrating that increased bone resorption can occur even when overall energy availability is maintained and systemic endocrine disruption is absent. The increase in β‐CTX, despite unchanged P1NP suggests that a reduction of bone mineral density may be possible even when individuals are in energy balance and not in a state of LEA. In contrast, hepcidin remained unchanged, supporting previous observations that a low‐carbohydrate high‐fat diet does not necessarily aggravate iron regulatory responses [14], although this contrasts with reports of elevated resting hepcidin in elite male race walkers [13] and exercising females [11]. Differences between studies may reflect variation in training load, exercise intensity, or biological sex. Taken together, these findings reinforce evidence that carbohydrate restriction may selectively influence bone turnover rather than broader endocrine homeostasis.

The reduction in circulating IGF‐1 (Figure 4), together with downregulation of ribosomal, sarcomeric, and extracellular matrix‐associated proteins (Figure 6C), suggests that LCA may induce an early anti‐anabolic skeletal muscle environment. Although this response may not represent a clinically anabolic suppression, it is noteworthy that it contrasts skeletal muscle responses evidenced with low energy availability. The proteomic signature observed here suggests prioritization of substrate utilization flexibility over translational and structural maintenance, with increased abundance of lipid transport and metabolism proteins accompanied by suppression of proteins linked to protein synthesis and contractile architecture, yet there was no substantial up regulation of mitochondrial proteins (Figure 6), as we had hypothesized. These findings are in line with our previous reports showing limited effects of signaling and gene expression related to mitochondrial biogenesis when training with LCA in energy balance or LCA in energy deficit over < 24 h [10, 17], and the increase in perilipin 2 in LCA are similar to what we have observed with the use of high‐energy high‐fat diets [41]. However, we have also shown an increase in intracellular lipid coating proteins with acute low energy availability—with collateral LCA, our findings as a whole represent a significant contrast to our recent proteomic analysis showing a coordinated and widespread mitochondrial protein network upregulation in males over 5 days in comparable experimental conditions [16]. This suggests that carbohydrate restriction alone may initiate only part of the molecular programme associated with energetic stress and substrate availability. Therefore, given that energy availability may play an important role in determining skeletal muscle phenotype shift towards an oxidative phenotype [16], considerations of training with low glycogen availability to upregulate markers of mitochondrial biogenesis [19], may have to contemplate also the acute exposure to reduced energy availability to maximize the adaptive signal to aerobic exercise.

These findings could have important implications for the periodisation of carbohydrate and energy intake around exercise training. Specifically, they support the concept that energetic stress, rather than carbohydrate availability alone, may augment skeletal muscle adaptive responses. While these data do not support the use of—and we do not advocate for—chronic or severe energy restriction, they raise the possibility that brief, controlled reductions in energy availability may contribute to the adaptive stimulus when appropriately integrated within a training programme. This notion is consistent with observations in elite cyclists, where free‐living training patterns are often characterized by relatively stable energy intake, resulting in transient reductions in energy availability on high training load days and compensatory increases during recovery or lower‐load periods [42]. Carbohydrate periodisation remains a central strategy to align substrate availability with training intensity and duration [20]; however, the present findings suggest that acute energy deficit may provide an additional, and currently underappreciated, stimulus for skeletal muscle adaptation. Nonetheless, these initial findings are limited to acute responses within short‐term frameworks. To validate this hypothesis, subsequent investigations must utilize longer periods and/or interventions that mirror the conditions of real‐world applied settings.

Several limitations should be acknowledged. First, the short intervention duration was designed to isolate early responses to carbohydrate restriction but may underestimate slower endocrine or tissue adaptations. Second, although crossover control strengthened internal validity, the modest sample size necessitates cautious interpretation of molecular outcomes, particularly proteomic findings. Third, these data were obtained in trained males under tightly controlled laboratory conditions and may not directly generalize to females, elite athletes, or prolonged field‐based training environments. Fourth, while we included a number of participants consistent with the majority of comparable experimental studies in this area [1, 16, 19] for the key parameters measured in this study, we cannot discard the possibility that a larger number of participants would have resulted in significant differences with a smaller magnitude of the effect of the intervention. Finally, whether these early molecular alterations translate into sustained functional consequences remains uncertain. However, the dissociation between preserved endocrine homeostasis and selective tissue‐level responses suggests that carbohydrate availability may independently influence adaptive pathways relevant to bone and muscle health in athletes.

In summary, carbohydrate restriction during 4 days under tightly controlled energy‐balanced conditions in trained males induces marked metabolic adaptation without reproducing the broad endocrine and skeletal muscle responses to training with low energy availability. Nevertheless, selective alterations in bone resorption, IGF‐1, and skeletal muscle proteomic signatures indicate that carbohydrate availability may independently modulate skeletal muscle phenotype, but not to the extent observed with concomitant energy deficit. These findings may help to refine current models of athlete energy deficiency by suggesting that carbohydrate availability alone is unlikely to drive systemic endocrine suppression, and that acute energy deficit could represent a stimulus that magnifies the skeletal muscle adaptation to aerobic exercise.

## Author Contributions

Conceptualization: J.L.A.; Data curation/software: E.M.‐L., Y.N., J.G.B., J.L.A.; Formal Analysis: E.M.‐L., Y.N., J.G.B., J.L.A.; Methodology: E.M.‐L., H.L.T., S.O.S., J.A.S.; Visualization: E.M.‐L., Y.N., J.G.B., J.L.A.; Funding acquisition: J.L.A.; Project administration: J.L.A.; Supervision: C.L.‐E., J.P.M., J.G.B., J.L.A.; Writing original draft: E.M.‐L., J.L.A.; Writing review and editing: E.M.‐L., H.L.T., Y.N., C.L.‐E., S.O.S., J.A.S., J.P.M., J.G.B., J.L.A.

## Funding

This project was funded internally by Liverpool John Moores University with a Seed Corn internal award to J.L.A.

## Conflicts of Interest

J.L.A. has received research funding from the Alliance for Potato Research and Education, The Society for Endocrinology (UK) and UK Research and Innovation. H.L.T. is employed as a scientist at Mondelez International. J.P.M. is a consultant to Science and Sport Ltd. S.O.S. is employed by Precision Hydration LtD as head of sports science. All human data collection and experimental procedures reported in this manuscript were conducted at Liverpool John Moores University (LJMU).

## Supporting information


**Figure S1:** Box plots of proteins in top‐left quadrant—Related to Figure 6. Box plots of 30 proteins with a significant interaction between group (LOW vs. NORM) and time (Day 0 to Day 4) (all *p* < 0.05). The box plot represents the interquartile range (IQR; 25th–75th percentile), with the horizontal line indicating the median. Whiskers extend to the minimum and maximum values within 1.5× IQR.
**Figure S2:** Box plots of proteins in bottom‐right quadrant—Related to Figure 6. Box plots of 27 proteins with a significant interaction between group (LOW vs. NORM) and time (Day 0 to Day 4) (all *p* < 0.05). The box plot represents the interquartile range (IQR; 25th–75th percentile), with the horizontal line indicating the median. Whiskers extend to the minimum and maximum values within 1.5× IQR.

**Table S1:** Statistical output of within‐subject two‐factor ANOVA in protein abundance associated to Figure 6.

## Data Availability

The data that support the findings of this study are available from the corresponding author upon reasonable request. The mass spectrometry proteomics data generated in this study have been deposited to the ProteomeXchange Consortium via the PRIDE [43] with the dataset identifier PXD077494 and DOI: 10.6019/PXD077494. Reviewers can access the deposited data by logging into the PRIDE website using the following details: Project accession: PXD077494, Token: OXcypX4yqjwz.
