# Supplementary material for: Low Carbohydrate Availability in Energy Balance Alters Bone Turnover and Muscle Proteomic Response With Limited Endocrine Disruption
Source: FASEB J. 2026 Aug 31;40(17):e72204. doi: 10.1096/fj.202602531R (PMC13527594; doi:10.1096/fj.202602531R)
Supplement: Supplementary file 1 [file FSB2-40-e72204-s002.docx]

**Figure S1 – Box plots of proteins in top-left quadrant – Related to Figure 6**

Box plots of 30 proteins with a significant interaction between group (LOW vs NORM) and time (Day 0 to Day 4) (all *P* < 0.050). The box plot represents the interquartile range (IQR; 25th–75th percentile), with the horizontal line indicating the median. Whiskers extend to the minimum and maximum values within 1.5× IQR.

**Figure S2 – Box plots of proteins in bottom-right quadrant – Related to Figure 6**

Box plots of 27 proteins with a significant interaction between group (LOW vs NORM) and time (Day 0 to Day 4) (all *P* < 0.050). The box plot represents the interquartile range (IQR; 25th–75th percentile), with the horizontal line indicating the median. Whiskers extend to the minimum and maximum values within 1.5× IQR.
